# Evaluation of Potential Factors Influencing the Dissemination of Multidrug-Resistant *Klebsiella pneumoniae* and Alternative Treatment Strategies

**DOI:** 10.3390/tropicalmed8080381

**Published:** 2023-07-26

**Authors:** Thando Ndlovu, Lebang Kgosietsile, Pako Motshwarakgole, Sizwe I. Ndlovu

**Affiliations:** 1 Department of Biological Sciences, Faculty of Science, University of Botswana, Private Bag UB, Gaborone 0022, Botswana; kgosietsilel@ub.ac.bw (L.K.); 201801713@ub.ac.bw (P.M.); 2 Department of Biotechnology and Food Technology, Doornfontein Campus, University of Johannesburg, Johannesburg 2028, South Africa; msizwe@uj.ac.za

**Keywords:** *Klebsiella pneumoniae*, antibiotic-resistant mechanisms, reservoirs, environment, global climatic variations, multidrug resistance, community-associated MDR

## Abstract

The increasing reports of multidrug-resistant *Klebsiella pneumoniae* have emerged as a public health concern, raising questions about the potential routes for the evolution and dissemination of the pathogenic *K. pneumoniae* into environmental reservoirs. Potential drivers of the increased incidence of antimicrobial-resistant environmental *K. pneumoniae* include the eminent global climatic variations as a direct or indirect effect of human activities. The ability of microorganisms to adapt and grow at an exponential rate facilitates the distribution of environmental strains with acquired resistant mutations into water systems, vegetation, and soil which are major intersection points with animals and humans. The bacterial pathogen, *K. pneumoniae*, is one of the critical-priority pathogens listed by the World Health Organization, mostly associated with hospital-acquired infections. However, the increasing prevalence of pathogenic environmental strains with similar characteristics to clinical-antibiotic-resistant *K. pneumoniae* isolates is concerning. Considering the eminent impact of global climatic variations in the spread and dissemination of multidrug-resistant bacteria, in this review, we closely assess factors influencing the dissemination of this pathogen resulting in increased interaction with the environment, human beings, and animals. We also look at the recent developments in rapid detection techniques as part of the response measures to improve surveillance and preparedness for potential outbreaks. Furthermore, we discuss alternative treatment strategies that include secondary metabolites such as biosurfactants and plant extracts with high antimicrobial properties.

## 1. Introduction

Antimicrobial resistance is generally described as the ability of microorganisms to survive in the presence of antimicrobial agents at concentrations typically sufficient to inhibit or kill them. It can further be explained as a multifactorial process that from a bacterial standpoint depicts an evolution in action, associated with continuous exposure to antibiotics and microbial genome plasticity [1,2,3]. Consequently, this evolution has led to the emergence of multidrug-resistant (MDR) and extremely drug-resistant (XDR) *Enterobacteriaceae* strains that are resistant to almost all antibiotics currently in use [4]. The spread of MDR *Klebsiella pneumoniae* is mostly associated with clinical settings where it is facilitated by factors such as closed space and spread between patients who are typically immunocompromised through what is termed, hospital-acquired infections. The increasing global climatic variations and the interconnectedness of the world have brought an increase in the community spread of these multidrug-resistant pathogens.

*Klebsiella pneumoniae* has been recognized as one of the multidrug-resistant pathogens of global concern and it is part of the ESKAPE (*Enterococcus faecium*, *Staphylococcus aureus*, *Klebsiella pneumoniae*, *Acinetobacter baumannii*, *Pseudomonas aeruginosa*, and *Enterobacter* species) organisms [5]. The *Klebsiella* genus is characterized by its Gram-negative, facultatively anaerobic, mostly capsulated, and non-motile bacteria that belong to the *Enterobacteriaceae* family [2,6]. *Klebsiella pneumoniae* is commonly associated with nosocomial infections such as urinary and respiratory tract infections as well as bloodstream infections [7,8]. It is a significant opportunistic pathogen mostly affecting individuals with weakened immune systems, such as those with cancer, diabetes, human immunodeficiency virus/acquired immunodeficiency syndrome (HIV/AIDS) as well as infants and the elderly [9]. This organism is particularly common in hospitals, where it can be spread from patient to patient through contact with contaminated surfaces or equipment such as catheters or ventilators [10]. Owing to its prevalence in hospital settings, *K. pneumoniae* is constantly exposed to antibiotics, in intensive care units (ICU) [11], maternity wards, and surgical theatres, thus subjecting it to constant selective pressure which leads to the occurrence of multiple genetic mechanisms conferring resistance to antibiotics [12].

On the other hand, environmental isolates of *K. pneumoniae* are often susceptible to antibiotics [13]. In non-clinical habitats, *K. pneumoniae* is ubiquitous in soil, surface water sources, plants, and the gastrointestinal tract of mammals, and environmental *K. pneumoniae* is not typically associated with human infections [13,14,15,16]. There is a major public health concern regarding the potential spread of community-associated MDR *K. pneumoniae* due to the rise in the number of reported cases. One of the possible considerations is that since *K. pneumoniae* is widespread in soil and water, the environment poses a potential reservoir for the infection of human beings and animals with these bacteria [13]. The emerging evidence suggests that some of the environmental isolates are identical to clinical isolates regarding their biochemical characteristics and virulence properties [14]. Global climate change is having a significant impact on the evolution of resistance in regular environmental *Klebsiella* species that are typically not pathogenic. Climatic variations lead to changes in temperature, precipitation, and other environmental factors that are creating new selective pressures for bacteria. These selective pressures are driving the evolution of new strains of *Klebsiella* that are resistant to antibiotics [17,18]. For example, intense precipitations will lead to increased runoff and inevitably higher levels of pollution in our surface and groundwater sources. Some of the pollutants that include antibiotics and heavy metals can then induce the expression of antibiotic-resistance genes and facilitate bacterial mutagenesis [18].

Considering the increased vulnerability of communities as they interact with multidrug-resistant pathogens and the possibility of future outbreaks, it is equally important to develop rapid and reliable detection and surveillance strategies as well as to develop alternative treatment antimicrobials. The implementation of rapid molecular-based detection methods has improved our preparedness for potential outbreaks. The detection of the 16S rRNA methylase, which belongs to the *armA* gene family, is an example of a reliable marker used in molecular detection methods for the rapid identification of MDR *K. pneumoniae* by gene sequencing and polymerase chain reaction (PCR) [19]. Nevertheless, information on the clinical significance of *Klebsiella* acquired from the environment is still lacking, especially in Sub-Saharan Africa.

In this review, we assess the increased prevalence of multidrug-resistant *K. pneumoniae* in communities and the environment, we assess the impact of global climatic variations in the spread of this pathogen resulting in increased dissemination to communities and the environment. Furthermore, we evaluate the progress in detection strategies to strengthen surveillance measures as well as alternative treatment measures that hold the potential for escaping the treatment barriers presented by multidrug resistance.

### 1.1. Klebsiella pneumoniae in Clinical Settings

*Klebsiella pneumoniae* is a notorious hospital-associated pathogen responsible for most bloodstream infections, urinary tract infections, pneumonia, and meningitis [7,20]. It is reported to spread from person to person in clinical settings, although not all carriers of *K. pneumoniae* develop the disease state [13]. Owing to its hospital association, *K. pneumoniae* is the dominant carbapenem-resistant *Enterobacteriaceae* globally [21], as well as the major extended-spectrum beta-lactamases (ESBL)-carrying pathogen responsible for nosocomial infections [2]. In a study conducted by Buys et al. [22] in a South African hospital, 83% of bloodstream infections were caused by ESBL-producing *K. pneumoniae* isolates with a 26% mortality rate. The study also revealed that 95% of the infections were hospital-acquired while 5% were community-acquired. Exposure to cephalosporins, pediatric ICU admission, and prolonged hospitalization were named as risk factors for these infections. *Klebsiella pneumoniae* possesses the ability to adhere to hospital equipment and this is thought to be what facilitates its distribution. All ESBL-producing isolates were resistant to ampicillin, cefotaxime, ceftazidime, and cefepime, but least resistant to amikacin and ciprofloxacin. Non-ESBL-producing isolates were, however, only 100% resistant to ampicillin and sensitive to the aforementioned antibiotics [22]. Major concerns are raised with the emergence and proliferation of carbapenem-resistant *K. pneumoniae* (CRKP) particularly because carbapenems are often administered as antibiotics of last resort [23]. Carbapenem-resistant Enterobacteriaceae (CRE) strains constitute a special challenge because they are resistant to β-lactams and there are few effective treatments for CRE-induced illnesses [23]. Taking this into consideration, the prevalence of risk factors for CRKP in hospitalized patients has been evaluated. Risk factors identified include previous hospitalization and prolonged stay in intensive care units (ICU). [24], previous use of carbapenems and β-lactam inhibitors [25], as well as subjection of patients to invasive procedures such as central venous catheterization [26,27]. Additionally, long-term care facilities are thought to be the major role players in the spread of MDR *K. pneumoniae* between clinical settings and community settings [28]. Al-Zalaban et al. [29] conducted a study that reported on the prevalence and trends of *Klebsiella pneumoniae* antibiotic resistance in King Fahad Hospital in Medina from 2014 until 2018. Out of a total of 15,708 isolates, 38.4% and 46.1% were resistant to imipenem and meropenem, respectively; third and fourth cephalosporins displayed resistance ranging from 57.5% to 77.8%. The research highlighted an increased resistance to beta-lactams as well as the emergence of resistance to carbapenems and alternative last-resort antibiotics such as colistin and tigecycline.

The development of antibiotic resistance in *K. pneumoniae* is a serious problem. Several measures can be put in place to treat infections or preventive measures for surveillance and to curb the spread of drug-resistant bacteria such as *Klebsiella* species. By working together, we can help to protect ourselves from this and other infections caused by antibiotic-resistant bacterial species.

### 1.2. Klebsiella pneumoniae in the Environment

*Klebsiella pneumoniae* is also found ubiquitous in non-clinical habitats such as the mucosal surface of humans and animals, vegetation, soil, and surface waters [30]. There is growing evidence that some of the environmental isolates of *K. pneumoniae* present identical genetic and phenotypic features to their clinical counterparts [15,31]. Although human pathogenic strains of *K. pneumoniae* are mainly associated with clinical settings, there is a noticeable shift in virulence oozing into the environmental isolates thus raising concerns about the factors driving this distribution of resistance factors. There is a growing concern that some strains of *K. pneumoniae* may be acquiring antibiotic-resistance genes from other bacteria in the environment [32]. In addition, the concurrent evolution and antimicrobial-resistant determinants acquisition by *K. pneumoniae* in the clinical settings and the environment has led to environmental habitats being considered as a possible reservoir for hyper-resistant *K. pneumoniae* [33]. The genetic determinants that specify resistance to different antimicrobial drugs are spread in strongly selective environments, with the presence of persistent antimicrobial residues in ecosystems significantly contributing to environmental pollution with resistant genes [34]. It is thought that environmental bacteria that generate and release antibacterial elements to influence microbial populations with which they compete for nutrients are a source of antimicrobial-resistant genes [35]. Additionally, Booth et al. [36] suggest that a high concentration of antimicrobials in the environment exists because of their stability.

Several studies have been conducted to understand the genetic similarities between the environmental and clinical *K. pneumoniae* isolates [15,31,33]. The environmental reservoirs of *K. pneumoniae* associated with human infections are not well known. In a study by Podschun et al. [14], they used hemagglutination assay and serotyped *K. pneumoniae* isolates obtained from surface water (from various streams, lakes, and baltic sea in Germany) by the capsular swelling method to check for the similarity of their virulence factors to those of clinical isolates. Their study determined that *K. pneumoniae* isolates obtained from surface water resembled clinical isolates in their expression of virulence factors, although their relevance to public health could not be established. Similarly, Struve and Krogfelt [15] also compared the virulence of environmental *K. pneumoniae* isolates from surface waters in Denmark to clinical isolates. Struve and Krogfelt [15] utilised counter-current immunoelectrophoresis for capsule typing, dot-blotting using radio-labelled DNA for the detection of adhesin genes and hemagglutination assays to test for the expression of fimbriae. Their study revealed that environmental isolates were as virulent as clinical strains as per their detection methods. Recently, Rocha et al. [31] conducted a comparative genomics study between environmental and clinically associated *K. pneumoniae* strains of distinct geographical locations. They found environmental strains that were closely related to those found in clinical settings.

Multidrug-resistant *K. pneumoniae* has previously been isolated from raw wastewater [6] and sewage plants [37]. This incidence reveals two scenarios, which include the exposure of clinical waste to the environment and possible active dissemination of antibiotic-resistance genes to aquatic environments and from there to agricultural settings and eventually to humans. In a study by Salifu et al. [38], ESBL-producing MDR *K. pneumoniae* has also been isolated in wastewater from a public healthcare facility in Nigeria, further justifying the assumption that water sources play a major role in the dissemination of disease-causing MDR *K. pneumoniae* in the environment. Additionally, Devarajan et al. [39] investigated the incidence of antibiotic resistance genes (ARGs) in a tropical river in Congo receiving hospital and urban wastewater. Although their study targeted the detection of *E. coli* and *Pseudomonas spp* genes, it still supported the notion that water sources could act as reservoirs for ARGs of *K. pneumoniae* and other *Enterobacteriaceae* species as their study revealed the presence of these genes in the said river.

Limited studies recognise *K. pneumoniae* as a food-borne pathogen that can utilize plants as reservoirs and find its way back to humans and animals [40]. Thus, various vegetables and food commodities have been identified as possibly harbouring this pathogen and these include carrots, spinach, cucumber, and tomatoes among others [41]. Animals are no exception, an extended-spectrum beta-lactamase (ESBL) blaCTX-M-IS, which is most prevalent in *K. pneumoniae* has since been found in humans. This inherently implies the possible transmission of *K. pneumoniae* from animals to humans because this ESBL is a zoonotic agent commonly applicable to animals [42]. A study in Germany identified multiple MDR-ESBL strains from companion animals including cats, dogs, and horses. The *K. pneumoniae* isolates from these animals were found to share the same characteristics as those from humans with regard to the presence of plasmid-encoded quinolone resistance genes, ESBLs, and carbapenemase OXA-48 [42]. This incidence of common *K. pneumoniae* genes between animals and humans is thought to be evidence of the transmission of MDR genes between humans and animals.

Overall, studies reporting on environment-acquired *K. pneumoniae* infections are based on a single event or case series. Currently, there is limited research on the estimation of incidences of community-acquired multidrug-resistant *K. pneumoniae*. Controversies arise when one must determine the significance of each reservoir in the spread of MDR *K. pneumoniae*. It can be argued that environmental reservoirs such as contaminated surface water sources can serve as primary sources of transmission and dissemination of multidrug-resistant bacterial strains. In contrast, the prolonged stay in hospital or frequent use of antibiotics by patients are also drivers of transmission. There is a need for understanding the interplay between the clinical and environmental reservoirs for the designing of effective prevention and control strategies [43,44,45]. Research is currently exploring the use of various techniques for the detection and identification of multidrug-resistant bacteria including *K. pneumoniae*, to unravel the complexities of the primary sources of dissemination [43]. In the developing world, details concerning the methods for full characterization of the bacterial isolates are limited, which is an important limitation for the reporting of multidrug-resistant microbial strains.

## 2. Factors Driving the Increasing Resistance of Community-Associated *K. pneumoniae*

The acquisition of resistance to a wide range of antibiotics by the typical *K*. *pneumoniae* is manifested through its resistance to treatments for simple infections such as urinary tract infections (UTIs) and life-threatening infections [46]. Different factors have been identified to influence the spread of multidrug-resistant *K*. *pneumoniae* strains. It is therefore critical to identify these resistance-causing factors and understand how treatments aimed at addressing them could curb antibiotic resistance [47]. Selected mechanisms of resistance by *K. pneumoniae* are briefly discussed below.

### 2.1. Antibiotic-Resistant Mechanisms of Klebsiella pneumoniae

The modes of antibiotic resistance in *K. pneumoniae* are encoded either intrinsically or acquired through mutation and resistance gene acquisition [48]. Several factors including the stress-induced acquisition of resistance determinants, altered membrane barrier functions, and promotion of biofilm formation can influence the adaptive response of *K. pneumoniae* [49]. Beta-lactam antimicrobials exemplify the most common therapeutic form available for the treatment of bacterial infections and as a result, bacteria continue to develop resistance to β-lactam antibiotics worldwide [50]. The consistent exposure to a variety of β-lactams has caused a dynamic, ongoing production and mutation of β lactamases, improving the bacteria’s response even against recently developed β-lactam antibiotics [50].

*Klebsiella pneumoniae* employs an enzymatic-resistant mechanism by producing enzymes known as extended-spectrum β-lactamases (ESBLs) [51]. The β-lactamases mainly include TEM, SHV and CTX-m types (Table 1) [50]. Ghenea et al. [52], detected the presence of all β-lactamase enzyme types in *K. pneumoniae* strains with the SHV being most common in all strains. These enzymes have a changed substrate profile because of amino acid substitution which allows for the hydrolysis of most cephalosporins [53]. The ESBLs typically confer resistance towards penicillins, first, second and third generation cephalosporins as well as aztreonam [54]. *Klebsiella pneumoniae* produces a zinc-dependent metalo-β-lactamase NDM-1 (Table 1), anchored to external membranes [55]. Strains producing the plasmid gene (blaNDM-1) encoded NDM-1 hydrolyze β-lactam antibiotics, except aztreonam [55].

The genes coding for TEM and SHV enzymes have a high mutation rate; as a result, there is an increase in the level of diversity of the enzyme types, thus increasing the range of antibiotic resistance [63]. A different class of enzymes includes the metallo- β-lactamases (MBLs) [59]. According to Kumarasamy et al. [59], genes encoding the MBLs in *K. pneumoniae* are found on plasmids, therefore, are easily transmitted to other microorganisms with the most common being imipenemase metallo-β - lactamases (IMP) and Verona-integron-encoded metallo-β- lactamases (VIM) (Table 1). A novel phenomenon is the chromosomal integration of usually plasmid-encoded ESBL genes [64]. *Klebsiella pneumoniae* lacks genes coding for chromosomal AmpC *β*-lactamase, which is an enzyme with the ability to inactivate aztreonam, all penicillin, and most cephalosporins and which is not susceptible to inhibition by most β-lactamases inhibitors. Alternatively, the acquisition of transmissible plasmids from other bacteria can lead to the overproduction of AmpC β-lactamase *K. pneumoniae* [65].

Changes in membrane permeability and drug flux can be influenced by variable expression and regulation of the efflux pumps [43]. The significant efflux pump is the resistance nodulated division (RND) (Table 1). The efflux systems of the RND type are known to accommodate a broad range of structurally unrelated molecules that can include most classes of antibiotics, as well as biocides, metabolic inhibitors, aromatic hydrocarbons, bile salts, and homoserine lactones linked to quorum sensing [66]. Furthermore, the cell-density-dependent expression of several virulence factors can be influenced by the activity of these efflux pumps. Padilla et al. [67] suggest that the AcrAB efflux system (Table 1) of *K. pneumoniae* may promote the release of the molecule(s) significant for the virulence of the microbe. According to Schneiders et al. [68], over expressions of the transcriptional regulator RamA or mutations in the AcrAB repressor (AcrR) are the two main contributors to the enhanced production of the AcrAB efflux pump in 19 fluoroquinolone-resistant *K. pneumoniae* strains. There is, however, a lack of understanding of the precise and direct contribution of AcrAB multidrug efflux pump’s contribution to *K. pneumoniae* resistance to other drugs [68].

An additional modification to the outer membrane (OM) aiding in the resistance of *K. pneumoniae* is the upregulation of capsule polysaccharide (CPS) production by altering penicillin-binding proteins (PBPs) [69]. This process decreases the affinity of the PBPs to the β-lactam antibiotics [70]. It is involved in the resistance to antimicrobial peptides, proteins (AP), and β-lactams [57].

### 2.2. Stress-Induced Acquisition of Antimicrobial Resistance in K. pneumoniae

Antimicrobial resistance mechanisms can be modulated by environmental factors such as temperature, pH, availability, and competition for nutrients [71]. According to the United Nations Framework, climate change is defined as a result of human activities (direct or indirect) as well as natural climatic variations over a period which leads to changes in the composition of the global atmosphere [72]. The effect of climatic variations on microscopic organisms is understudied, yet microorganisms form an important support system of the biosphere [17]. More important is the intersection of rising temperatures as a result of global climate change and the rise in antibiotic resistance [18]. For instance, temperature variations have historically played a major role in the evolution and adaptation of life on Earth. The pathogen, *K. pneumoniae*, provides a good model of adaptation upon exposure to antibiotics and thermal stress [73]. Higher temperatures affect microbial growth and gene expression, which may lead to changes in bacterial susceptibility to antibiotics [71]. This means that microbial resistance to antibiotics can evolve as a collateral effect due to higher temperatures. Although these physiological and phenotypic changes may be temporal responses, they provide enough time for the bacterial population to finetune their antimicrobial resistance mechanisms to withstand the selective pressure posed by higher temperatures [71].

A study by McFadden et al. [74] showed that for every 10% increase in temperature, there was a 2.2% increase in the antibiotic-resistance profile of *K. pneumoniae*. Studies conducted by McGough et al. [75] and Pärnänen et al. [76] have also reported that higher temperatures were a critical factor in increased antimicrobial resistance. One of the possible mechanisms of the intersection between climatic variations and increased antimicrobial resistance can be explained using the well-known fitness trade-off phenomenon in bacterial strains that have acquired antimicrobial resistance mutations [71]. For instance, one of the susceptibility-determining factors in *K. pneumoniae* strains is the expression of two major porins (Ompk35 and Ompk36) which provides a gateway for the diffusion of hydrophilic molecules such as β-lactams. The lack or reduced expression of these porins in *K. pneumoniae* strains typically confers resistance to β-lactams, as they cannot penetrate [70]. The conferred resistance comes at a cost of reduced metabolic and cellular functions resulting in slower growth rates [73]. Evidence suggests that the fitness cost may be alleviated under certain conditions such as higher temperatures while antibiotic resistance is conserved [71]. This confirms the reports that antimicrobial resistance may evolve as a collateral effect of thermal stress. These findings can be extrapolated to explain the increased antimicrobial resistance in environmental *K. pneumoniae* isolates coinciding with increased thermal stress as a consequence of global climatic variations.

### 2.3. Other Global Warming Factors Facilitating Microbial Infections and Dissemination of Antimicrobial Resistance

Global warming is thought to exacerbate the incidence of antimicrobial resistance worldwide. These two major health crises are seen as interconnected public health challenges [77]. Warm temperatures allow the atmosphere to hold water, which consequently leads to severe storms and precipitation. High precipitation rates lead to flooding which is linked to the destruction of human and animal shelters as well as water and food sources. The rise in temperatures due to climate catastrophe has been linked to AMR simultaneously increasing in humans, animals, and the environment [74,75]. Flooding, for instance, damages municipal sewage systems leading to the overflow of sewage into water reservoirs such as rivers, oceans, and lakes [78]. Consequently, this water leads to the distribution of pathogenic microorganisms to surface waters and vegetation [79]. Evidence from flooding incidences in the United States of America such as New Orleans in 2005 (Hurricane Katrina) and Houston in 2017 suggest an increase in the distribution of infection-disease causing pathogens such as *E. coli, K. pneumoniae,* and *Pseudomonas* species in flooded areas with some of the bacteria carrying antibiotic-resistant genes [80]. Henriot et al. [81] assessed the prevalence of three bacteria contamination markers in water: *E. coli*, *Pseudomonas,* and *K. pneumoniae* in 16 floodplain wetlands and three rivers in Massit, France by sampling on a monthly interval for a one-year period. In addition, they also assessed water physico-chemical characteristics. The abundance of *K. pneumoniae* was noted during warm summer seasons suggesting its distribution during high river flows and favoured distribution in warm waters [81]. Furthermore, the drought that comes with climate change often reduces the availability of clean water and proper sanitation, forcing people to use low-quality water for consumption, agriculture, and other domestic uses. This scenario/action leads to the contamination of farm produce by the use of contaminated water with antimicrobial-resistance genes or pathogenic bacteria during irrigation. This increases the potential exposure of humans to pathogens through ingestion of contaminated produce or direct contact with contaminated water [78]. Thus, ESBL-producing pathogens have been isolated from farm produce [82,83]. Nevertheless, San Lio et al. [77] stated that while the relationship between pathogens and temperature increase does not imply climate change will result in increased AMR; it implies that antimicrobial-resistant pathogens are increasingly under selective pressure at higher temperatures. Additionally, several studies have revealed that warmer climates have an effect on concentrations of biocides and heavy metals in soils and water bodies as well as their uptake by bacteria [84,85]. Consequently, this causes antimicrobial resistance through co-resistance mechanisms.

Overall, studies reporting on the environment acquired *K. pneumoniae* infections are based on a single event or case series. In the developing world, details concerning the methods for full characterization of the bacterial isolates are limited, which is an important limitation for the reporting of multidrug-resistant microbial strains.

## 3. Methods for Detection of *Klebsiella pneumoniae* and Its Resistant Genes

The increasing presence of *Klebsiella pneumoniae* in environmental sources such as water, soils, and agricultural produce, increases the risk they pose as reservoirs for animal and human infections. Not neglecting the clinical settings, where *K. pneumoniae* is one of the most reported nosocomial causes of various infections. The accurate identification of microorganisms, including *K. pneumoniae*, is essential for accurate diagnosis of diseases that can facilitate the correct prescribing of medication for treatment as well as the keeping records of disease outbreaks associated with microbial infections.

Commonly used traditional methods of bacterial characterisation rely on phenotypic recognition of the disease causative organism using various techniques. These methods include identification by use of culturing on various culture media, colony morphology, Gram-staining, and biochemical analyses [86]. There are drawbacks of using these methods, with the major drawback being that they can only be used for culturable microorganisms and that certain bacterial strains could exhibit unique biochemical characteristics that do not fit into patterns that have been considered specific for any known genus and species. Therefore, phenotypic identification methods such as the analytical profile index (API) systems are however the most utilised for the rapid presumptive identification of microbial species. It is important to note that a given bacterial species genome is complex and the biochemical characteristics in the identification systems may not accurately indicate the organism’s metabolism. Microbial phenotypic properties can be unpredictable at times as their expression is dependent upon changes in the environmental conditions such as the substrate used for its growth, temperature, or pH fluctuations [87]. Despite the above-mentioned drawbacks of phenotypic methods, they are still considered important in clinical laboratories for the identification of medically important bacterial species and remain the method of choice for primary identification because of their cost-effectiveness.

Molecular-based detection methods that include PCR amplify specific selected target DNA of the organism of interest. Ribosomal DNA (rDNA), more specifically the 16S (small subunit) rDNA genes have been used for studies at the sub-generic level, but such studies were deemed not appropriate by Boyer et al. [88] as the 16S rDNA genes were found to be highly conserved [89]. Probes were then designed to target the 16S-23S internal transcribed spacer (ITS) [88]. This 16S-23S internal transcribed spacer is genetically variable and species specific when compared to the 16S or 23S rRNA genes [89]. Primers designed by Liu et al. [89] to target the 16S-23S ITS of *K. pneumoniae* were employed for assessing their specificity. The benefit of using PCR is its high specificity and DNA can be targeted with specific primers, out-performing culture-based methods. The PCR is an excellent technique for the rapid detection of pathogens, including those that are difficult to culture [90].

To better understand the potential contribution of the environment as a vehicle of drug-resistant *Klebsiella pneumoniae*, a novel real-time polymerase chain reaction (PCR) method, the ZKIR (*zur-khe* intergenic region) assay, was developed by Barbier et al. [44] to detect multidrug-resistant and virulent *K. pneumoniae* in environmental samples. Complete specificity and sensitivity were observed, with the technique being sensitive enough to detect a single *K. pneumoniae* bacterium in 5 g of soil more than what could be observed using the culture-based method [44]. Similarly, Rodrigues et al. [91] conducted a multicentric study that characterized food isolates genotypically. The use of zur-khe intergenic region (ZKIR) in the quantitative PCR (qPCR) technique was implemented for the detection of *K pneumoniae* in chicken, meat, and salad leaves [91]. Compared to the culture method, the ZKIR quantitative PCR revealed high percentages of the multidrug-resistant *K. pneumoniae*.

Molecular detection via PCR remains the reference standard in most studies, with less information available on that of routine labs identifying the microorganisms from ill individuals, especially in Sub-Saharan Africa. Ali et al. [92] made use of a single-tube multiplexed PCR to screen for *K. pneumoniae* isolates harbouring the *magA* genes. The genes were analysed by restriction fragment length polymorphism (RFLP) assay of PCR which demonstrated the presence of the *magA* gene in multidrug-resistant clinical isolates of *K. pneumoniae*. However, Hrabák et al. [93] proposed the addition of matrix-assisted laser desorption/ionization time-of-flight mass spectrophotometry (MALDI-TOF-MS), and spectrophotometric methods as reference methods alongside molecular tests.

Moreover, Kundu et al. [94] compared two methods for the typing of *K. pneumoniae* isolates; one based on the genotype, enterobacterial repetitive intergenic consensus-polymerase chain reaction (ERIC-PCR) and protein profiling (MALDI-TOF). Different clustering patterns were observed between the methods, with MALDI-TOF revealing 6 (G1–G6) distinct groups while ERIC-PCR revealed 40 (E1–E40) distinct groups [94]. This communicates the importance of using other methods alongside molecular methods because the phenotype expressed may not be a true representation of the genotype. Therefore, due to the rapid increase in the emergence of multidrug-resistant microorganisms, which include *K. pneumoniae,* has given rise to infections that are responsive only to a limited consortium of last-resort drugs. Of further concern is that the prospects for the development of new, effective drugs are limited [95]. The development of new strategies which includes the use of alternative antimicrobial compounds is thus a priority. Such compounds such as phytochemicals and biosurfactants could either replace antibiotics (due to their various antibacterial mechanisms) or be used in conjunction with antibiotics.

## 4. Alternative Control Strategies for Antibiotic-Resistant *Klebsiella pneumoniae*

The rising prevalence of multidrug-resistant *Klebsiella pneumoniae* in hospital and environmental settings leaves health practitioners with fewer treatment options. Owing to this, efforts are currently being made on research in alternative antimicrobials which complement antibiotic therapy and help combat the further development and spread of resistance in the future [96]. Accordingly, particular focus has been awarded to biological control strategies due to their low resistant frequencies, environmentally friendly, cost effective, and self-sustaining abilities [97]. The review further highlights the alternative control strategies including plant extracts and microbially synthesised secondary metabolites such as biosurfactants against antibiotic-resistant *K. pneumoniae*.

### 4.1. Biosurfactants

Biosurfactants are amphiphilic compounds with hydrophobic and hydrophilic moieties, naturally produced by certain microbial species in distress. Groups of these compounds include glycolipids, lipoproteins, phospholipids, neutral lipids, and polymeric compounds [98,99]. They are known to be eco-friendly, biodegradable, and non-toxic putting them at an advantage compared to their synthetic counterparts. These compounds have been reported to have multiple applications in bioremediation, nanotechnology industries, and biomedicine [100]. Of more interest to this review is their potential application toward alleviating antimicrobial drug resistance as explored by various researchers. Biosurfactants produced by bacteria are most promising because of their antibacterial, antiviral, and antifungal properties. Ndlovu et al. [98] investigated the antimicrobial activity of biosurfactant extracts produced by *Bacillus amyloliquefaciens* and *Pseudomonas aeruginosa* against various Gram-positive and Gram-negative bacteria including *Klebsiella pneumoniae* (Table 2). The results showed significant antibacterial activity against two clinical antibiotic-resistant *K. pneumoniae* isolates used as test strains. Additionally, Zampolli et al. [101] and most of the studies reviewed reported that biosurfactants were effective against various *Escherichia coli* strains (*E. coli*). It is therefore implied that these compounds could be effective against *K. pneumoniae*, which falls under the same family of *Enterobacteriaceae* with *E. coli*.

### 4.2. Plant Extracts

Plant remedies have been used over the years and their action on microorganisms is a subject of interest in the search for new antibacterial therapies which are shown by phytochemicals. Phytochemicals are naturally occurring compounds found in plants, which exhibit a range of biological activities that include antibacterial activity. Some phytochemicals that have been shown to have antibacterial activity against *Klebsiella pneumoniae* include flavonoids, glycosides, gallic acid, tannins, phenolic acids, amongst others as listed in Table 2.

The chromatographic based methods such as gas chromatography-mass spectrometry (GC-MS) analysis have revealed that plant extracts are generally categorized into three broad classes, namely phenolic compounds, terpenes, and alkaloids, that all display antimicrobial activity [104,112]. The antibacterial activity of plant extracts has been reported against various Enterobacteriaceae inclusive of the MDR *Klebsiella pneumoniae*. Gufe et al. [105] investigated the efficacy of herb–herb combinations against MDR *K. pneumoniae*, acetone extracts of *Myrothamnus flabellifolius* were observed to have the most inhibition wherein their combination with *Ximenia Caffra* extracts demonstrated a potent synergistic antimicrobial activity against the clinical *K. pneumoniae* strain. In a study conducted by Cock and Vuuren [106], polyphenolic compounds (Coumarins) from *Prangos hulusii* root extracts are suggested to be active agents with antimicrobial properties against a reference standard and clinical strain of *K. pneumoniae*. No specific mode of action of the phytochemicals was reported. Plant extracts may manifest their antimicrobial effects not only by killing the microorganism but by also affecting crucial steps in the pathogenesis [113]. For example, Paula-Ramos et al. [114] observed a reduction in the *K. pneumoniae* biofilm when exposed to extracts of *Phlox paniculate* and suggested that the monoterpenes found in the extracts (Table 2) can alter membrane permeability by affecting the exchange of essential substances, leading to a metabolism collapse. In addition, Ibrahim et al. [109] suggested that since extracts from *Moringa peregrina* have antibacterial activity against both Gram-negative and positive bacteria, they may act on the protein structure or at a transcription level, and not among cell wall structure. Cock and Vuuren [106] demonstrate the antibacterial effects of a methanoic bark extract of *Syzygium cordatum* with a composition of phytochemicals such as tannins and phytosterols that contribute to the antimicrobial properties of the extract. It could be noted that different constituents of plant crude extracts act at different sites and in doing so contribute to the overall activity of the extract [113]. This reduces the likelihood of the development of antimicrobial resistance.

## 5. Conclusions

*Klebsiella pneumoniae* causes various types of infections that include pneumonia, urinary tract infections, and bloodstream infections. However, in recent years, there has been an increase in the antibiotic-resistant strains of *K. pneumoniae* that are causing complicated infections that include sepsis and death. The antibiotic resistance could be due to several factors, which include the overuse and misuse of antibiotics to treat infections, thus selecting for resistant *K. pneumoniae* strains. Other factors include the acquisition of genes that confer antibiotic-resistant genes from closely related bacterial species through horizontal gene transfer or conjugation. The rise in antibiotic-resistant *K. pneumoniae* infections is a public health concern and there is a need for techniques to study the mechanisms and detection of these resistant strains. Research has shown that the extended-spectrum beta-lactamases contribute to resistance towards a wide range of antibiotics, which include penicillins, cephalosporins, and carbapenems. Several methods are utilised for the detection of antibiotic resistance, which includes the disk diffusion assay, minimum inhibition concentration, and recently, genomic analysis. Genomic analysis has been shown to be more reliable and has accurately identified *K. pneumoniae* in infected individuals as well as outside a hospital setting. Several arguments are consistent with the involvement of extra-hospital reservoirs of *K. pneumoniae* in the occurrence of community-acquired infections. However additional studies are needed for a better estimation of the incidence of community-acquired *K. pneumoniae* infections, especially in Sub Saharan Africa, and a better understanding of the mechanisms of interactions between the different potential reservoirs and humans. Moreover, there is ongoing research on bioprospecting for alternative antimicrobial compounds that could be used to control the spread of antibiotic-resistant *K. pneumoniae*, including the use of phytochemicals and bacterial secondary metabolites such as biosurfactant compounds.

## Figures and Tables

**Table 1:** Antibiotic resistance mechanisms associated with *Klebsiella pneumoniae*.

Antibiotic (s)	Resistance Mechanism (s)		Location	Examples	Reference
β-lactams	Target altering	PBP	C	PBP3 (ftsl)	[56]
	Permeability modification	OMP	C	Ompk35 and Ompk36	[57]
	Efflux pumps	RND	P	AcrAB ad OqxAB	[57]
	Class A	ESBL	P	CTX-M, SHV-5, SHV-1, TEM-10, VEB	[57]
		Carbapenemase	Tn3-type transposon	KPC-1	[58]
		β-lactamase (Cephalosporinase)	P	CphA, IMP, SIM, VIM Class C	[57]
	Class B	metallo-β-lactamase	P	IMP, VIM, NDM-1	[59]
	Class D	ESBL	C & P	OXA-11, OXA-2, OXA-7, OXA-9, OXA-10, OXA-12	[57]
		Carbapenemase	P	OXA-48, OXA-51, OXA-181, OXA-237	[60]
Quinolone	Point mutation	DNA gyrase	P	gyraA and gyraB	[57]
		DNA topoisomerase	P	parC and parE	
Amynoglycosides	Enzymatic	AME	I	aac(6’)-lb-cr, aac(3’)-lid, aadA, aadB,	[61]
			P	aph	[62]
	Target mutations	RMTases	P	armA, rmt	[57]
Colistin	Point mutation	Inactivation of Pmrb	P	mcr-1	[57]
		Mutations in crrB fluroquinolones	P	mcr1.2	
Phenicols	Point mutation	Rifampin		catA, catB, cml, floR	[57]
Trimethoprim	Enzymatic	dihydrofolate reductase	I	dfrA12, sul1	[61]
Tigecycline	Efflux pumps	RND	P	rpsJ	[57]

I—integron; P—plasmid; C—chromosome; AAC—Acetyltransferases; AME—aminoglycoside-modifying enzymes; APH—phosphotransferases; CTX-M—Cefotaximase-Munich; IMP—imipenem metallo-β-lactamase; NDM—New Delhi metallo-β-lactamase; OMP—outer membrane protein; OXA—oxacillinase; P—plasmid; PBP—penicillin-binding protein; PER—*Pseudomonas* extended resistance; RMTases—16S RNA methylase; RND—resistance-nodulation division; SHV—sulfhydryl variant; SIM—Seoul imipenem metallo-β-lactamase; TEM—Temoniera; VIM—Verona integrin-encoded metallo-β-lactamase.

**Table 2:** Representative of alternative antimicrobial agents that have been reported on *K. pneumoniae*.

Type of Biological Control	Source of *Klebsiella pneumoniae* Isolates	Producer Species	Class of Compound	Mode of Action	Reference
Biosurfactant	Clinical and Environmental	*Pseudomonas aeruginosa*	Rhamnolipids	Cell membrane disruption	[98,102,103]
	Clinical	*Bacillus amyloliquefaeciens*	Lipopeptides	Not specified	[98]
Plant extracts	ATCC 13883	*Syzgium Cordatum* bark	Tannins, sterols, triterpenoids	Not specified	[104]
	Clinical	*Myrothamnus flabellifolius* and *Ximenia caffra*	Flavonoids, glycosides, gallic acid, tannins	Not specified	[105]
	ATCC 4352	*Phlox paniculata*	Triterpene and monoterpene	Biofilm reduction	[106]
	Clinical	*Plumbago indica* root	Plumbagin	Not specified	[107]
	Clinical and ATCC 4352	*Prangos hulusii* root	Polyphenolic compounds (Coumarins)	Not specified	[108]
	Not specified	*Moringa peregrina*	Unspecified	Protein or DNA structure	[109]
	Clinical	*Clitoria Ternatea* root	Unspecified	Not specified	[110]
	ATCC 27736	*Indigofera oblongifolia* leaf	Phenolic and flavonoid	Not specified	[111]

ATCC—American Type Culture Collection.

## Data Availability

Not applicable.
